# Anxiety on the lockdown resolution

**DOI:** 10.1192/j.eurpsy.2021.758

**Published:** 2021-08-13

**Authors:** S. Ajmi, S. Hentati, R. Masmoudi, R. Sellami, I. Baati, I. Feki, J. Masmoudi

**Affiliations:** 1 Psychiatrie A, hospital University Hedi Chaker, sfax, Tunisia; 2 Psychiatry A, hedi chaker hospital, Sfax, Tunisia; 3 Psychiatrie “a” Department, Hedi Chaker Hospital University -Sfax - Tunisia, sfax, Tunisia; 4 Psychiatry “a” Department, Hedi Chaker Hospital University -Sfax - Tunisia, sfax, Tunisia

**Keywords:** Anxiety, COVID19, lokdown resolution, tunusia

## Abstract

**Introduction:**

Lockdown due to the management of infectious diseases such as corona virus disease affect mental health. We would think that with the end of the lockdown due to the corona virus pandemic, the feeling of regaining freedom and movement would be good for our morale.

**Objectives:**

Through this servery, we examine the prevalence of anxiety symptoms in the Tunisian population face to the end of lockdown.

**Methods:**

The survey was conducted using the online anonymous questionnaires and distributed through social networks from 24 April to 23 May 2020(which was considered the end of the lockdown in Tunisia). It included socio-demographic questions and participants’ experience of SARS-CoV-2related stressful events (A member of your family was suspected of having Corona virus /someone you know had Corona virus‘s symptoms /You were quarantined). Anxiety symptoms were evaluated with the Hospital anxiety and depression scale-anxiety (HADS-A)

**Results:**

Our study included 80 participants: 71.3% female and 42.5% married. The mean age of the participants was 29.30 years (SD = 8.72). The mean HADS-A score was 8.03 (SD=2.938) (maximum=16 minimum=1). Two-thirds of the participants exhibited anxiety symptoms (66.3%) with 1.3 % reported moderate severe anxiety symptoms. Anxiety was correlated with age and gender (p=0.013, p=0.027).

**Conclusions:**

Our results suggest that in this early phase of the COVID-19 lockdown resolution we can already observe its fundamental impact on anxiety.

